# Fabrication of Spherical Titania Inverse Opal Structures Using Electro-Hydrodynamic Atomization

**DOI:** 10.3390/molecules24213905

**Published:** 2019-10-30

**Authors:** Jong-Min Lim, Sehee Jeong

**Affiliations:** Department of Chemical Engineering, Soonchunhyang University, 22 Soonchunhyang-ro, Shinchang-myeon, Asan-si, Chungcheongnam-do 31538, Korea; jsh951004@naver.com

**Keywords:** colloidal crystal, inverse opal, electro-hydrodynamic atomization, photonic ball, titania

## Abstract

Spherical PS/HEMA opal structure and spherical titania inverse opal structure were fabricated by self-assembly of colloidal nanoparticles in uniform aerosol droplets generated with electro-hydrodynamic atomization method. When a solution of PS/HEMA nanoparticles with uniform size distribution was used, PS/HEMA nanoparticles self-assembled into a face-centered cubic (FCC) structure by capillary force with the evaporation of the solvent in aerosol droplet, resulting in a spherical opal structure. When PS/HEMA nanoparticles and anatase titania nanoparticles were dispersed simultaneously into the solution, titania nanoparticles with relatively smaller size were assembled at the interstitial site of PS/HEMA nanoparticles packed in the FCC structure, resulting in a spherical opal composite structure. Spherical titania inverse opal structure was fabricated after removing PS/HEMA nanoparticles from the spherical opal composite structure by calcination.

## 1. Introduction

Photonic bandgap is a phenomenon that originated from periodically arranged materials having different refractive indices. It can be controlled by changing the structure of periodicity and the refractive index of constituent material. Yablonovitch et al. have demonstrated the fabrication of 3D photonic crystal having a photonic bandgap in the microwave region [1]. Since then, there have been various studies on the fabrication of 3D photonic crystal structures [2,3,4,5,6,7]. With a bottom-up approach, colloidal self-assembled structures have been widely adopted to make 3D photonic crystal structures due to their simplicity and cost-effectiveness. As the solvent of colloidal dispersion evaporates slowly, nanoparticles with uniform size distribution are self-assembled into a hexagonal crystal lattice (i.e., face-centered cubic (FCC) structure) to form an opal structure. In addition, an inverse opal structure with more robust photonic bandgap can be prepared by filling interstitial sites of the opal structure using materials with high refractive index and removing colloids having a hexagonal crystal lattice [3,5,6,8].

One of the main issues in the fabrication of 3D colloidal photonic crystals (e.g., opal and inverse opal structures) is the control of their shape and size in a reproducible manner. In order to control size and shape, various studies have prepared spherical 3D colloidal crystals using uniform-sized droplets as confined geometries [6,7,8,9]. Electro-hydrodynamic atomization method, also known as electrospray, has been developed for large-scale production of spherical 3D colloidal crystals. Moon et al. have demonstrated electro-hydrodynamic atomization for large-scale production of spherical polystyrene opal structures and spherical silica inverse opal structures [10]. Since they used water as a solvent, an additional process was required for the evaporation of the solvent. In addition, spherical titania inverse opal structures could not be fabricated due to poor dispersion stability of titania nanoparticles in aqueous solution. Hong et al. have prepared spherical silica opal structures without an additional solvent evaporation process using ethanol as a solvent in electro-hydrodynamic atomization [11]. They also used crosslinked polystyrene nanoparticles in toluene for electro-hydrodynamic atomization. However, spherical structures with irregularly packed colloidal crystal shells and hollow cores were fabricated due to the extremely fast evaporation rate of toluene.

In this study, we used poly [styrene-co-(2-hydroxyethyl methacrylate)] nanoparticles (PS/HEMA nanoparticles) with enhanced stability in ethanol to fabricate spherical PS/HEMA opal structures and spherical titania inverse opal structures by electro-hydrodynamic atomization. Without the additional evaporation process, we could prepare compact spherical PS/HEMA opal structures regularly packed in an FCC structure. When a mixture of PS/HEMA nanoparticles in ethanol and anatase titania nanoparticles in methanol is used for electro-hydrodynamic atomization, titania nanoparticles of relatively small size were assembled at the interstitial site of PS/HEMA nanoparticles packed in the FCC structure, resulting in a spherical opal composite structure. After calcination to remove PS/HEMA nanoparticles, spherical titania inverse opal could be fabricated. Since the electro-hydrodynamic atomization method can rapidly prepare a large amount of spherical PS/HEMA opal structures and spherical titania inverse opal structures, it can expedite the commercial application of spherical opal and spherical inverse opal structures in various areas, including reflective mode display, photo catalysis, solar cell electrode materials, and analytical systems [12,13,14,15,16,17].

## 2. Results

### 2.1. Characterization of Monodisperse PS/HEMA Nanoparticles and Titania Nanoparticles

**Figure 1:**
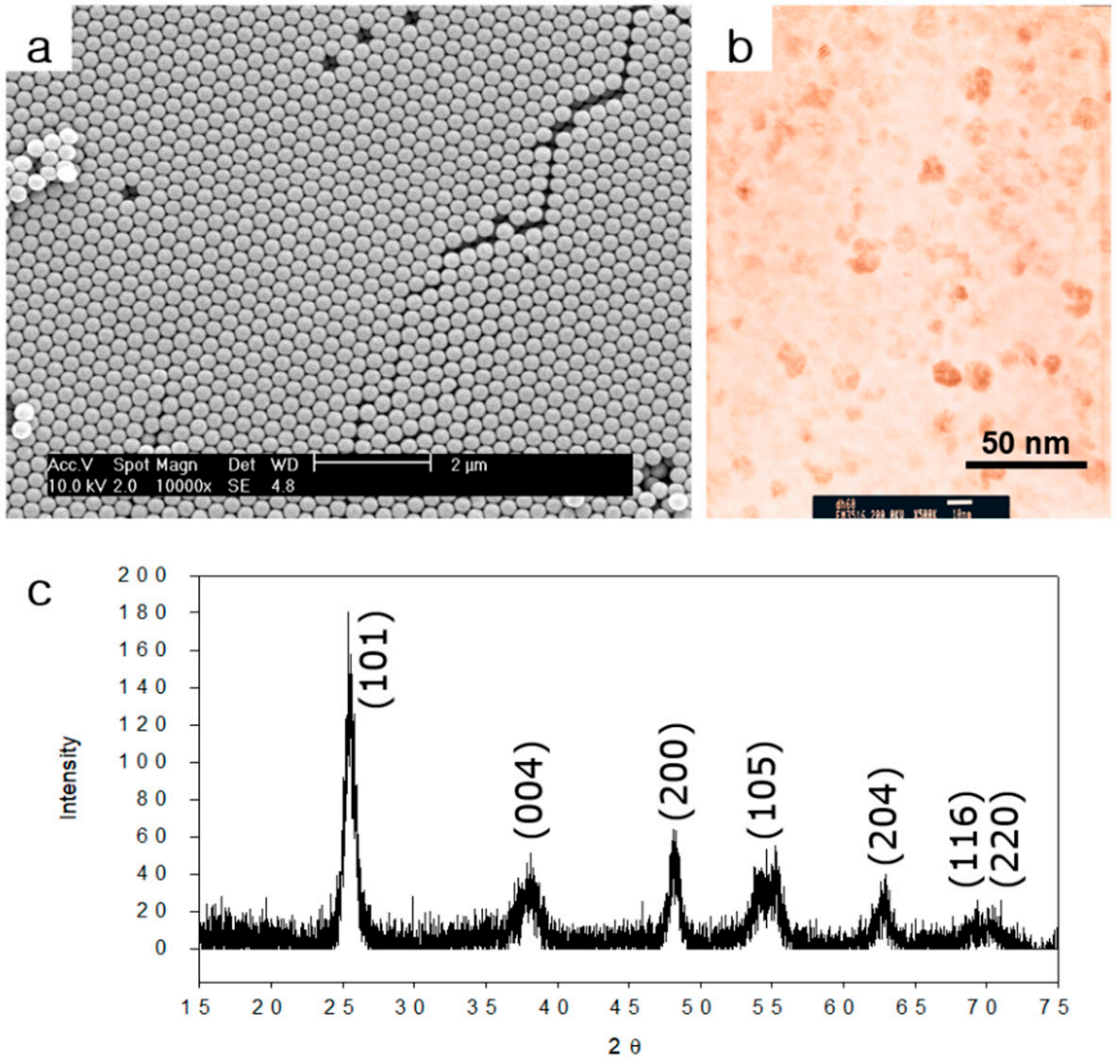
(**a**) SEM image of PS/HEMA latex nanoparticles. (**b**) TEM image and (**c**) x-ray diffraction data of titania colloidal nanoparticles.

Figure 1a shows an SEM image of monodisperse PS/HEMA nanoparticles. PS/HEMA nanoparticles were hexagonally packed (i.e., FCC structure), confirming uniform size distribution of PS/HEMA nanoparticles. Figure 1b shows a TEM image of titania nanoparticles. These titania nanoparticles had an anatase phase based on power x-ray diffraction as in Figure 1c.

### 2.2. Preparation of Uniform Aerosol Droplets Using Electro-hydrodynamic Atomization

AC electric field in the range of 1.2–1.8 kV/mm intensity with frequency of 1–5 kHz was used to maintain stable Taylor cone jet mode. The frequency of the AC electric field did not significantly affect the size of aerosol droplets because the frequency range (i.e., 1–5 kHz) was high enough [11]. 

### 2.3. Fabrication of Spherical PS/HEMA Opal Structures Structures

**Figure 2:**
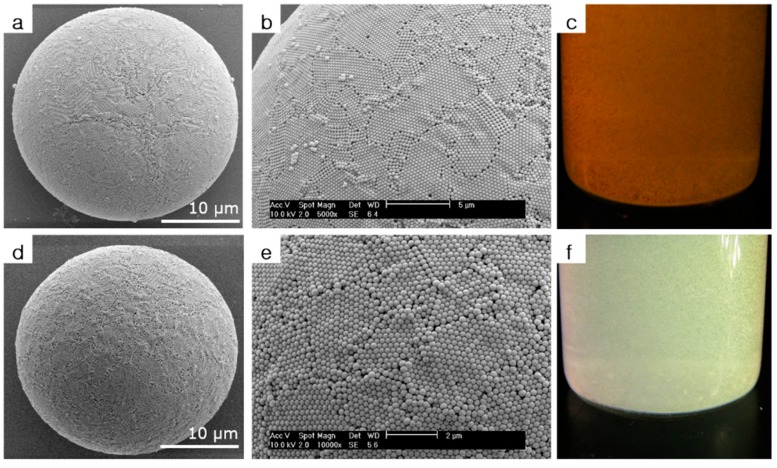
(**a**,**b**) SEM images and (**c**) digital camera image of spherical opal structures made of 280 nm-sized PS/HEMA nanoparticles. (**d**,**e**) SEM images and (**f**) digital camera image of spherical opal structures from 210-nm-sized PS/HEMA nanoparticles.

Figure 2a–c and Figure 2d–f show SEM and digital camera images of the spherical opal structure consisting of 280 and 210-nm-sized monodisperse PS/HEMA nanoparticles, respectively. As shown in Figure 2a,d, spherical opal structure could be fabricated as the solvent evaporated. As shown in magnified SEM images (Figure 2b,e), monodisperse PS/HEMA nanoparticles self-assembled into a hexagonal crystal lattice, showing the (111) plane of the FCC structure. 

### 2.4. Fabrication of Spherical Titania Inverse Opal Structures

**Figure 3:**
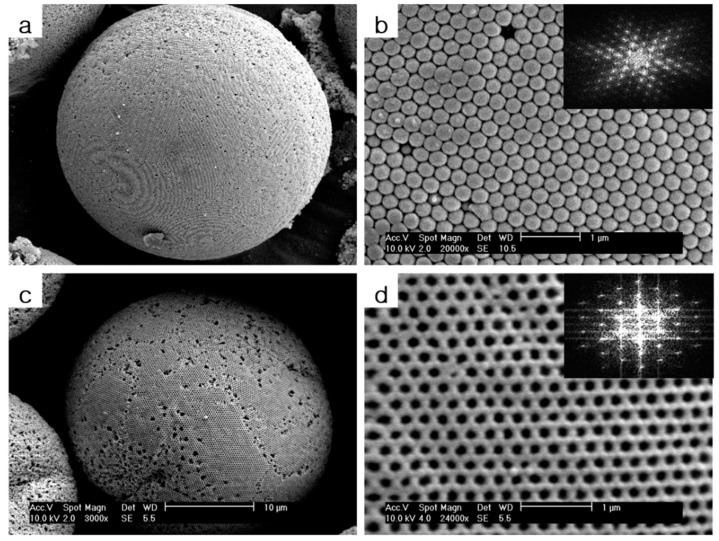
(**a**,**b**) SEM images of spherical opal composite structures consisting of PS/HEMA nanoparticles and titania nanoparticles. (**c**,**d**) SEM images of spherical titania inverse opal structure. Inset shows the fast Fourier transform (FTT) of the SEM images.

Since both PS/HEMA nanoparticles and anatase titania nanoparticles could make stable colloidal dispersion in the mixture of ethanol and methanol at a 3:1 volumetric ratio, we could use mixed colloidal dispersion for electro-hydrodynamic atomization. Figure 3a,b show SEM images of spherical opal composite structures consisting of PS/HEMA nanoparticles and anatase titania nanoparticles. As shown in Figure 3c,d, spherical titania inverse opal structures could be obtained after removing PS/HEMA nanoparticles by 500 °C calcination. The inset of Figure 3b,d shows the fast Fourier transform (FTT) of the SEM images.

## 3. Discussion

The size of the spherical opal structure was determined by the size of the aerosol droplet produced by electro-hydrodynamic atomization and amounts of nanoparticles in an aerosol droplet. Since the concentration of nanoparticles in the solution was kept constant, uniform aerosol droplets should be stably generated to produce spherical opal structures with uniform size distribution.

Uniform aerosol droplets of several tens to several hundreds of micrometers in size could be generated in a controlled manner using electro-hydrodynamic atomization method. When colloidal dispersion was injected into the capillary needle at a constant flow rate using a syringe pump, droplets were formed at the end of the capillary. When gravity became greater than restoring surface tension, the droplet detached from the capillary and dripped through the ring electrode. In the electro-hydrodynamic atomization method, AC electric field was applied to a stainless capillary needle and a ring electrode. Because AC electric field deforming electrical tangential stress was applied to the meniscus, when the electric field increased, the size of the dripping droplet became smaller due to electrical tangential stress known as dripping mode. When the applied electric field exceeded the threshold value, the meniscus became a cone shape known as Taylor cone jet mode [18]. In the Taylor cone jet mode, droplets with uniform size distribution could be generated stably. As electric field strength further increased, multiple unstable jets were formed at the end of the stainless steel capillary known as multi-jet mode [10,11].

The solvent in aerosol droplets evaporated while droplets passed the cylindrical plastic tube. As the solvent evaporated, nanoparticles in the droplet gradually got closer and began to self-assemble by capillary force. When nanoparticles were brought into contact with each other, nanoparticles were fixed to a spherical opal structure by van der Waals force. The self-assembly and fixation process could be completed in a few seconds after droplet generation by electro-hydrodynamic atomization.

Since the sizes of titania nanoparticles were much smaller than those of PS/HEMA nanoparticles, titania nanoparticles were assembled at the interstitial site of hexagonally packed PS/HEMA nanoparticles. The periodicities of the structures in Figure 3b,d are 239 nm and 224 nm, respectively. The periodicity of the structure was decreased by about 6.3% by the calcination. Because of the defects in Figure 3b, there are slight differences between the average diameter of PS/HEMA nanoparticles and the periodicity of the structure.

A large amount of spherical PS/HEMA opal structure could be obtained rapidly using the electro-hydrodynamic atomization method. As shown in Figure 2c,f, spherical PS/HEMA opal structures were dispersed in water in order to demonstrate optical properties. Reflected diffraction colors could be controlled depending on the size of nanoparticles constituting the spherical opal structure. Since the surface of the spherical opal structure was composed of the (111) plane of the FCC structure, light corresponding to the bandgap of the photonic crystal was reflected. The bandgap position in wavelength (λ) could be estimated by Bragg’s law for the (111) plane of the FCC structure: [2,19]

(1) $$\lambda =2dn_{eff}={\left(\frac{8}{3}\right)}^{0.5}Dn_{eff}$$

where d was the spacing of the (111) plane, $n_{eff}$ was the effective refractive index, and *D* was the diameter of constituent nanoparticles. The $n_{eff}$ could be obtained by

(2) $$n_{eff}=\left\{\phi_{p}n_{p}^{2}+(1-\phi_{p})n_{m}^{2}\right\}$$

where $\phi_{p}$ was the volume fraction of nanoparticles, and $n_{p}$ and $n_{m}$ were refractive indices of nanoparticles and matrix, respectively. The bandgap position (λ) could be estimated from the Bragg’s law using the volume fraction of nanoparticles in the FCC structure ($\phi_{p}$ = 0.74), refractive index of PS/HEMA particles ($n_{p}$ = 1.59), and refractive index of water ($n_{m}$ = 1.33). For spherical opal structures composed of 280 and 210-nm-sized PS/HEMA nanoparticles, bandgap positions (λ) estimated from the Bragg’s law were 698 nm and 523 nm, respectively. Since spherical PS/HEMA opal structure could be fabricated in large quantities using the electro-hydrodynamic atomization method, bandgap positions (λ) can be visually confirmed from the digital camera images shown in Figure 2c,f.

The bandgap position (λ) of the spherical titania inverse opal structure could be estimated from Bragg’s law using the refractive index of anatase phase titania ($n_{m}$ = 2.5) and the refractive index of water ($n_{p}$ = 1.33). Here, we assumed that 74% of the unit cell structure was occupied by water for the case of spherical inverse opal structure. When 280-nm-sized PS/HEMA nanoparticles were used to make the spherical titania inverse opal structure, the bandgap was located in the near infrared region (i.e., λ = 783 nm). The bandgap position could be controlled by changing the size of PS/HEMA nanoparticles used to fabricate spherical titania inverse opal structures. The bandgap position can also be controlled by changing the refractive indices of materials used to make spherical inverse opal structures and solvents used to disperse the spherical inverse opal structure.

## 4. Materials and Methods

### 4.1. Chemicals

All chemicals and solvents were reagent grade and used without further purification. Styrene (99%) was purchased from Kanto chemical (Tokyo, Japan), and 2-hydroxyethyl methacrylate (reagent grade) was purchased from Sigma-Aldrich (St. Louis, MO, USA). Potassium persulfate (98%) as the initiator was purchased from Kanto chemical (Tokyo, Japan). Titania nanoparticles in methanol dispersion (DH 60) were obtained from Nissan chemicals (Tokyo, Japan). Ethanol (≥ 99.9%) was purchased from Merck (Kenilworth, NJ, USA).

### 4.2. Synthesis of Monodisperse PS/HEMA Nanoparticles

Monodisperse PS/HEMA nanoparticles were synthesized through batch type surfactant-free emulsion copolymerization in aqueous medium. Detailed synthesis procedures can be found elsewhere [20]. Aqueous PS/HEMA nanoparticle dispersion was dried on a piece of silicon wafer and coated with gold for observation using a field emission scanning electron microscope (FE-SEM, XL305FEG, Philips (Amsterdam, The Netherlands)).

### 4.3. Fabrication of Aerosol Droplets Using Electro-hydrodynamic Atomization

PS/HEMA nanoparticles were re-dispersed in ethanol prior to electro-hydrodynamic atomization. To make titania inverse opal, PS/HEMA in ethanol dispersion and titania in methanol dispersion were mixed at 3:1 volumetric ratio. Vortex mixer (Maxi mix Ⅱ, Thermo Scientific (Waltham, MA, USA)) and ultrasonic cleaner (EW-08893-21, Cole-parmer (Vernon, IL, USA)) were used for re-dispersion and the mixing process.

**Scheme 1:**
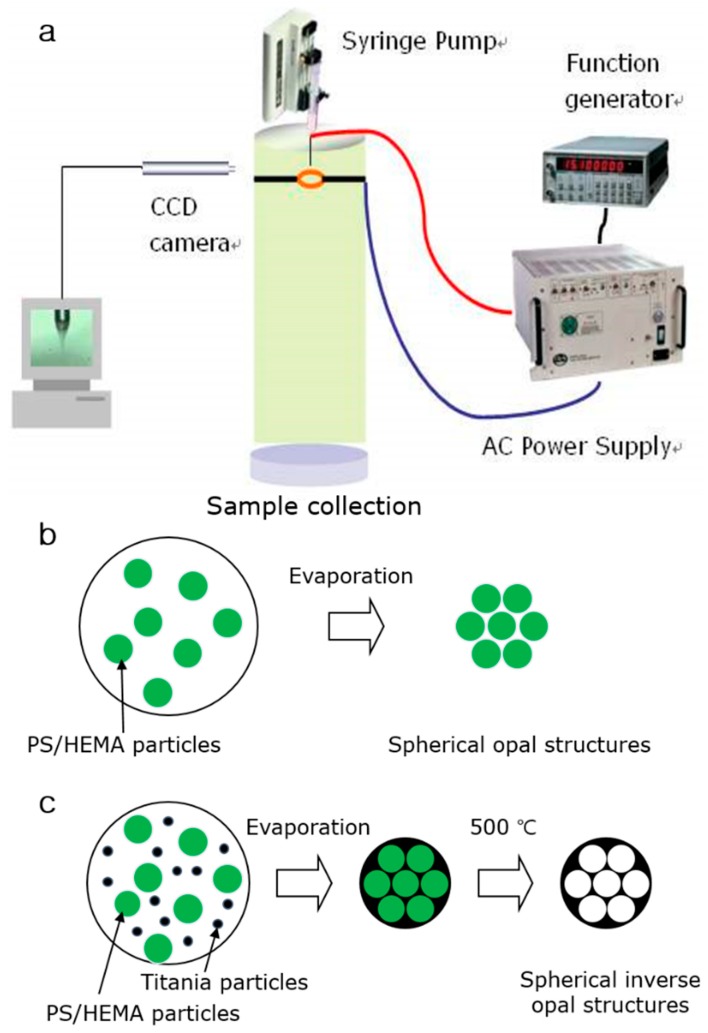
(**a**) Schematic of experimental set up of electro-hydrodynamic atomization for generating uniform droplets. Schematics of self-assembly processes of (**b**) spherical PS/HEMA opal structures and (**c**) spherical titania inverse opal structures.

Aerosol droplets with uniform size distribution were prepared by using the electro-hydrodynamic atomization method. The schematic of the experimental set up for electro-hydrodynamic atomization is illustrated in Scheme 1a. The apparatus for electro-hydrodynamic atomization consists of a colloidal dispersion injection part, an electric field control part, a meniscus observation part, and a colloidal self-assembled structure collection part. In the colloidal dispersion injection part, colloidal dispersion was introduced to a disposable 1-mL syringe which was connected to a stainless capillary needle (metal hub needle, Hamilton (Reno, NV, USA)). The disposable syringe was loaded to a syringe pump (KDS 100, KD Scientific (Holliston, MA, USA)) to maintain a constant flow rate of 0.5 mL/h. In the electric field control part, an arbitrary function generator (DS345, Stanford Research Systems (Sunnyvale, CA, USA)) and a high voltage power amplifier (20/20B, Trek Inc. (Lockport, NY, USA)) were connected to a stainless capillary needle and a ring electrode with circular hole of 1 cm in diameter. The shape of the meniscus formed on the capillary was changed with AC electric field. The AC electric field was maintained in the range of 1.2–1.8 kV/mm with 1–5 kHz frequency for stable generation of droplets in Taylor cone jet mode [18]. In order to precisely observe the shape, a CCD camera with a ring-shaped illuminator was used in the meniscus observation part. At the bottom of the ring electrode, there was a 90-cm-long cylindrical plastic tube for evaporating solvent in aerosol droplets. When the solvent in the aerosol droplet was completely evaporated as shown in Scheme 1b, PS/HEMA nanoparticles inside an aerosol droplet self-assembled to form spherical PS/HEMA opal structures. In addition, when PS/HEMA nanoparticles and titania nanoparticles were used simultaneously as shown in Scheme 1c, titania nanoparticles with smaller size were self-assembled at the interstitial site of hexagonally-packed PS/HEMA nanoparticles. As the solvent evaporated, spherical opal composite structures were formed as shown in Scheme 1c. Spherical opal structures and spherical opal composite structures could be collected by placing a glass petri-dish under the cylindrical plastic tube. As shown in Scheme 1c, spherical titania inverse opal structures could be obtained by removing PS/HEMA nanoparticles from the spherical opal composite structures via 500 °C calcination for about 8 hours using a muffle furnace (PK9712150030-9, Isuzu (Tokyo, Japan)). Spherical PS/HEMA opal structures and spherical titania inverse opal structures were observed using a FE-SEM (XL305FEG, Philips (Amsterdam, The Netherlands)).

## 5. Conclusions

Uniform aerosol droplets could be produced by the Taylor cone jet mode of the electro-hydrodynamic atomization method using an AC electric field. As the solvent evaporated, PS/HEMA nanoparticles self-assembled in droplets to create a spherical opal structure consisting of PS/HEMA nanoparticles. When PS/HEMA nanoparticles and anatase titania nanoparticles were simultaneously used, spherical opal composite structure could be prepared. After removing PS/HEMA nanoparticles by 500 °C calcination, spherical titania inverse opal structure could be fabricated. Bragg’s law was used to estimate the bandgap position (λ) of the spherical PS/HEMA opal structure and the spherical titania inverse opal structure. Since spherical opal structures could be prepared rapidly in large quantities using the electro-hydrodynamic atomization method, the bandgap position of spherical PS/HEMA opal structures could be visually confirmed from digital camera images. The bandgap position could be controlled simply by changing the size of PS/HEMA nanoparticles. The spherical opal structures and the spherical titania inverse opal structures produced by the electro-hydrodynamic atomization method are of practical significance for various applications, including reflective mode display, photo catalysis, solar cell electrode materials, and analytical systems.
